# Exploring an Applied Ecological Model of the Effects of Household, School, and Community Environments on Adolescent Mental Health in Japan

**DOI:** 10.3390/ijerph192416820

**Published:** 2022-12-14

**Authors:** Nagisa Mori, Azusa Arimoto, Etsuko Tadaka

**Affiliations:** 1 Public Health and Welfare Bureau, Nagoya 460-0002, Japan; 2 Department of Community Health Nursing, Graduate School of Medicine, Yokohama City University, Yokohama 236-0004, Japan; 3 Department of Community and Public Health Nursing, Faculty of Medicine, Graduate School of Health Sciences, Hokkaido University, Sapporo 060-0812, Japan

**Keywords:** mental health, mood and anxiety disorders, vitality, adolescent, household environment, school and community environment, ecological model, secondary analysis, covariance structure

## Abstract

Adolescent mental health is an urgent global public health issue and is affected by household, school, and community environments. However, few studies, and none in Japan, have used applied ecological models to identify environmental factors that affect adolescent mental health. This study aimed to examine an applied ecological model of sequential association between household, school, and community environmental factors and their effects on adolescent mental health in Japan (ECO-AM model). This was a secondary analysis of data from the 2013 Japanese Comprehensive Survey of Living Conditions. Participants were 893 adolescents aged 12–14 years and their household heads living in Japan. Data for 728 adolescents were analyzed after excluding participants with missing values (valid response rate: 81.5%). Screening using the six-item Kessler Psychological Distress Scale demonstrated that 33.8% of adolescents had mood and anxiety disorders. Covariance structure analysis yielded a model with strong goodness-of-fit that described associations between mood and anxiety disorder and vitality, and household, school and community environments. The explanatory variables accounted for 36% of mood and anxiety disorder scores. The study emphasizes the importance of the relationship between different environments and suggests that a better understanding of environmental factors would help support adolescent mental health.

## 1. Introduction

Mental health in adolescents is an urgent global public health issue. Mental health problems affect 10–20% of children and adolescents worldwide [1]. The World Health Organization [2] has defined mental health as “a state of well-being in which the individual realizes his or her own abilities, can cope with the normal stresses of life, can work productively and fruitfully” (p. 47). The consequences of not addressing adolescent mental health problems extend into adulthood, impairing both physical and mental health and limiting opportunities to lead a fulfilling adult life. Promoting psychological well-being and protecting adolescents from adverse experiences that may affect their potential to thrive are therefore essential for both adolescent well-being and adult physical and mental health [3]. Hale and Viner [4] studied a representative sample of adolescents in England. They found that adolescent health strongly predicts academic attainment and unemployment, even after controlling for childhood attainment, adult health, and sociodemographic factors, through mediators that include social exclusion, school behavior, truancy, substance use, and long-term absence. Adolescent mental health problems are also associated with suicidal behavior [5,6] and use of substances, such as cannabis and tobacco [4,7,8]. Half of adult patients with mental health disorders had an onset age of under 15 years [9,10] but most mental health problems in adolescents are undetected and untreated [3]. Preventing mental health difficulties and promoting mental health are two of the most important issues in adolescent health. However, research on mental health in adolescence is more limited in Asia than in Western countries. Most studies among Japanese adolescents have used school-based sampling [11,12], which limits their generalizability.

Mood and anxiety disorders are a leading cause of illness and disability among adolescents [3]. Hyakutake et al. [11] and Ojio et al. [12] showed that 20–30% of adolescents experience psychological distress. In Japan, approximately 14,000 adolescents have mood disorders, neuroses, stress-related disorder, or somatoform disorders, including anxiety disorders [13]. Mood and anxiety disorders are common mental health problems and strongly affect adolescent mental and physical well-being. They can lead to problems such as cannabis use [7], smoking [8], and suicidal behavior [5,6]. Studies of adolescent mental health, therefore, need to assess both illness and well-being.

Vitality is another important aspect of adolescent mental health status. Vitality is an individual’s subjective experience of energy and activity [14]. The General Well-Being Index includes vitality [15]. The 36-item Short Form Health Survey (SF-36), a measure of health-related quality of life, includes fatigue as a component of vitality [16]. Health-related quality of life is a multidimensional concept that includes domains related to physical, mental, emotional, and social functioning. One study found that vitality fully mediated the relationship between hope and physical health, social relationships, and environment, and partly mediated the association between hope and psychological health among 101 adult primary care patients [17]. Adolescent vitality can be defined as an expression of physical, mental, emotional, and social functioning. It therefore provides the energy for daily activities, including schoolwork, club activities, lessons, and play [18]. Headache has been reported as a factor related to physical and social functioning and quality of life in adolescents [19,20]. Chronic daily headaches are associated with a substantial reduction in quality of life, particularly physical functioning, bodily pain, vitality, and social functioning domains, among the general population [19]. Bohman et al. [20] demonstrated that somatic symptoms, such as headache and tiredness, are common in adolescent depression. Waza et al. [21] found a strong correlation between vitality and depression in a sample of 660 Swedish young people aged 14–20 years. Several studies [20,21,22,23] suggest that mood and anxiety disorders and vitality are important aspects of mental health status in adolescence. King et al. [23] studied a national sample of 21,993 grade 6–10 students in Canada and emphasized that mental health status can be fully determined with two dimensions: subjective well-being and psychopathology. They also suggested that people need good mental health to function well, including realizing their abilities, working productively, and maintaining healthy relationships [23]. Adolescent mental health assessment should, therefore, examine both mental illness and vitality as components of well-being.

In recent years, educational programs to promote mental health have focused on enhancing the abilities (e.g., help-seeking, problem-solving ability, and coping) of individual adolescents, and have become widespread worldwide. However, some studies have indicated that these educational programs have small effects [24] or are not effective [25]. Previous studies of environmental factors also suggest a need to clarify the background factors (e.g., sociodemographic and environmental factors) associated with support to improve mental health [26,27]. However, these studies only investigated high school students and adults and did not fully assess the influence of the household environment, which is important for adolescents. To promote adolescent mental health, it is, therefore, important to nurture individual abilities, such as help seeking, problem solving, and coping, and also to improve adolescents’ environments, including households, schools, and communities [28].

Bronfenbrenner’s most recent ecological theory [29] posits that relationships between developmental environments and the dynamics that occur within them are fundamental in understanding the influences on adolescent mental health. A central tenet of this theory is that individual development is affected by the ongoing qualities of adolescents’ social settings and the interactions between settings in the developmental environment (e.g., the head of the household, family, friends, schools, and community) [30,31]. The investigation of mental health in adolescents must focus on mental illness and vitality—a component of well-being [23]—because mood and anxiety disorders and vitality are related [20,21], Therefore, in this study, adolescent mental health was assessed by the primary outcome of mood and anxiety disorders and the secondary outcome of vitality. 

It is important to consider environmental factors in adolescence within a comprehensive framework of family, school, and community environments. Piko et al. [32] found that socioeconomic status (SES), parental support, and optimism are directly and indirectly associated with depression in high school students (aged 14–20 years) in Hungary. They highlighted the need to consider both socioeconomic differences in social networks and levels of optimism in depression prevention and treatment. However, previous studies [31,33] were conducted within the school environment rather than within a comprehensive framework of household, school, and community environments. These previous studies [32,33] measured indicators of SES, such as income, but did not examine other important aspects of the household environment, such as mood and anxiety disorders in the head of the household. More research is needed to evaluate the complex interactions between variables that moderate and mediate the association between low SES and negative adolescent outcomes. An ecological model is needed to explore the complex effects of the interactions between household, school, and community environmental factors on adolescent mental health. 

The household environment is an important predictor of adolescent mental health [34,35,36,37,38]. Parental mental illness negatively affects the mental health of adolescents both genetically and environmentally [34]. Single parenting [35,36], parental attachment [39] and low SES [23,37,38] are also all risk factors for mood and anxiety disorders in adolescence. In a study of adolescents (15–17 years old) in Korea, Lee, and Kwon [35] demonstrated that adolescents who lived with a single parent or a non-parental guardian were more likely to be depressed than those living with both parents. Muzi et al. [39] demonstrated that more positive peer attachment mediated 61% of the effect of the parental secure attachment on withdrawal/depression, revealing an indirect effect of parental attachment on withdrawal through peer attachment. King et al. [23] found that mentally healthy young people were most likely to live with both parents and report their family as being wealthy among a national sample of 21,993 grade 6–10 students in Canada. Depression prevalence is positively associated with parental unemployment and living in family structures other than with parents [37]. In addition, in a study of adolescents (12–15 years old) in Japan, Hyakutake et al. [11] demonstrated that having a parent with depressive symptoms was significantly associated with students’ depressive symptoms.

The school and community environments are important predictors of adolescent mental health [38,40,41]. Support resources, such as family, friends, and teachers [23,38,40,41], school climate [28,31], community safety [33], and high regional suicide rates [35] are related to adolescent mental health. A systematic review showed that adolescents, aged 10–19 living in low SES homes and communities, have a greater risk of experiencing negative psychosocial outcomes through effects on parental depression, parental conflict, negative parenting, and adolescent ability to cope [28]. A cross-sectional study demonstrated that adolescents, aged 15 years from disadvantaged backgrounds, who reported strong connections to their parent, school, and neighborhood, were more likely to report fewer depressive symptoms. Parent and school connectedness were also significantly related to teen anxiety [41].

**Figure 1:**
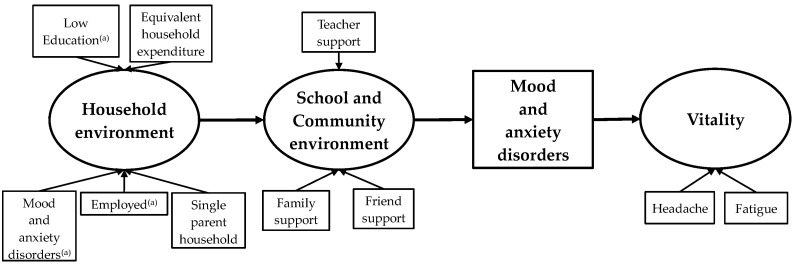
Applied ecological model of effects of household, school, and community environments on adolescent mental health. Measurement items for mood and anxiety disorders and error variance are not shown. ^(a)^ Household head.

We constructed a sequential relationship model of mental health and hypothesized that household environment would be the primary latent factor and the school and community environment the secondary latent factor. This was based on the results of our previous study that indicated a need to clarify the background factors (e.g., sociodemographic and environmental factors) associated with support to improve mental health [26,27]. We predicted that the household environment, as part of the basic nurturing environment (which influences adolescents’ knowledge, attitudes, and skills), would affect adolescent mental health. We also predicted that the school and community environment, as part of the socialization environment (which forms adolescent experiences and relationships with friends, teachers, and the community), would affect adolescent mental health. To examine the primary latent factor of household environment, we assessed several household SES variables, including single parent household, household head in employment, household head with low levels of education, and equivalent household expenditure. We also assessed the psychosocial indicator of whether the household head had mood and anxiety disorders. To examine the secondary latent factor of the school and community environment, we assessed the following observed variables: family support, friend support, and teacher support. Household environment was defined as the environment with respect to parents. We also included family support as part of the school/community environment, because families include siblings (at school) and grandparents and relatives (beyond the household, in the community). Figure 1 shows the conceptual model that was developed for this study. The objective was to examine an applied ecological model of the effects of the sequential association between household, school, and community environmental factors on adolescent mental health in Japan (ECO-AM model).

## 2. Materials and Methods

### 2.1. Study Design

This was an observational, cross-sectional study that used data from the Comprehensive Survey of Living Conditions (CSLC) conducted by the Japanese Ministry of Health, Labour and Welfare [42]. The purpose of the CSLC is to evaluate the basic living conditions of residents in Japan (e.g., health, medical care, welfare, pensions, and income) and to obtain data useful for planning national policies on health, labor, and welfare. The CSLC started in 1986, and a large-scale survey has been conducted every three years since. This study used data from the most recent survey (2013) to conduct secondary analysis. Permission to use these data under the 1947 Statistics Act was obtained from the Japanese Ministry of Health, Labour and Welfare.

### 2.2. Study Participants

**Figure 2:**
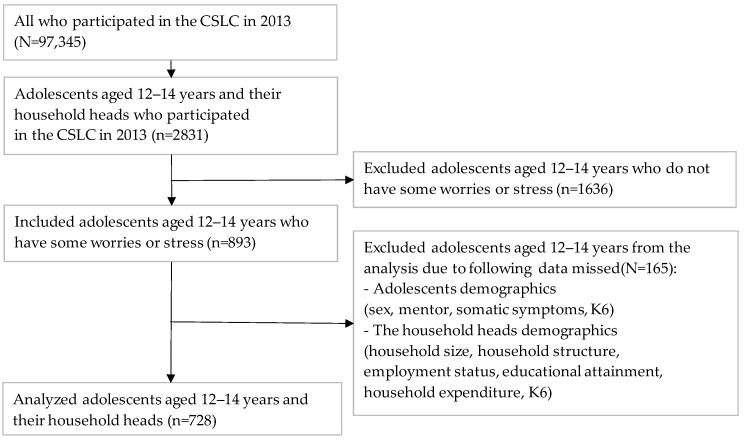
Flowchart of participant selection process.CSLC: the Comprehensive Survey of Living Conditions; K6: six-item Kessler Psychological Distress Scale.

Participants were adolescents aged 12–14 years (n = 2831) and their household heads, whose data were drawn from the CSLC dataset (n = 97,345). The CSLC participants are randomly selected from the 5530 National Census districts of Japan, covering approximately 300,000 households. The study target participants were 893 adolescents aged 12–14 years who answered “Yes” to the single question “Do you currently have any worries or stresses?”. Data for 728 of the 893 adolescents and their household heads (valid response rate: 81.5%) were analyzed after excluding participants with missing data values (n = 165). Figure 2 is a flowchart showing the participant selection process.

### 2.3. Measures

#### 2.3.1. Mental Health

The Japanese version of the six-item Kessler Psychological Distress Scale (K6) [43] was used to assess mood and anxiety disorders in adolescents and their household heads. The K6 measures how frequently participants have experienced symptoms of psychological distress during the previous month. The six questions are rated on a five-point Likert-type scale: 0 = none of the time, 1 = a little of the time, 2 = some of the time, 3 = most of the time, and 4 = all the time. Higher total scores indicate a high likelihood of mood and anxiety disorders. Previous studies [43,44,45,46] have defined K6 scores of 5–12 as indicating moderate disorder and K6 scores of ≥13 as indicating severe disorder. The CSLC does not include a measure of vitality. Therefore, we used the presence of several somatic symptoms (e.g., fatigue, headache, and irritability) to assess the vitality associated with mood and anxiety disorders. The presence of each symptom was indicated by a score of 1 and its absence by 0.

#### 2.3.2. Household Environment

To examine household environment, we measured household structure, employment status of the household head, educational attainment of the household head, equivalent household expenditure, and mood and anxiety disorders of the household head. The household structure included categories as follows: a couple and unmarried children only, three-generation household, single parent and unmarried children only, and other households, such as siblings only or uncles or aunts and children. Previous studies indicate a relationship between mental health and household structure among adolescents. Household structure was therefore categorized as single parent and unmarried children only (scored as 1) or the other three categories (scored as 0). Single parent and unmarried children refers to one parent living in the household with children who have not yet left home to start families of their own. In Japan, such families accounted for just 6.4% of all families in 2019. This is because in Japan, divorce rates are relatively low and children usually leave home as soon as they have completed schooling. The employment status of the household head was categorized as employed (1) or not employed (0). Educational attainment of the household head included categories such as primary or junior high school, completion of high school, vocational school, 2-year college/technical college, university, or graduate school. Education was categorized as primary or junior high school (1) or higher (the other five items) (0). In the context of the compulsory education system in Japan, this indicated low (1) or high (0) levels of education. Equivalent household expenditure was calculated by dividing household expenditure per month by the square root of the household size [47]. Household expenditure was the amount of expenditure during May of the survey year.

#### 2.3.3. School and Community Environment

To examine the school and community environment, we measured who the adolescents consulted about their worries and stress. We used this because it was the only question that included content about school and community, i.e., mentioning teachers and friends. Participants were asked, “To whom do you talk about your problems and stress? (Multiple answers permitted)”. Those who consulted family members were considered to have family support, categorized as Yes (1). Alternatively, no family support was indicated by No (0). Those who consulted friends/acquaintances were considered to have the “support of friends” as Yes (1) or No (0), and those who consulted school teachers were considered to have the “support of teachers” as Yes (1) or No (0). Adolescents who used the counseling services of public institutions, received counseling from a doctor at a hospital or clinic, or consulted someone other than the above were considered to have “other support” as Yes (1) or No (0). Adolescents who responded, “I would like to consult someone but have nobody to ask” or “I would like to consult someone but do not know where to go” were considered to have “no social support”. We classified responses to each category as Yes (1) or No (0).

#### 2.3.4. Demographic Variables

The gender of adolescents was classified as boy (1) or girl (2). Household size was classified as 2, 3, 4, 5, or ≥6 people.

### 2.4. Statistical Analysis

First, the simple questionnaire item totals for each component of the conceptual model (ECO-AM model; Figure 1) and the demographic variables were calculated. Items scored by less than 5% of respondents (n = 728), such as “no social support” and “stomachache”, were excluded from the analysis to ensure representativeness. Next, Spearman’s correlations among the variables were used as the basis for modeling using the covariance structure analysis. Structural Equation Modeling (SEM) with maximum likelihood was used to identify the optimal model by ascertaining the path directions and standardized estimates of each path, and by calculating the comparative fit index (CFI) and root mean square error of approximation (RMSEA) of the whole model. As a sub-analysis, the CFI and RMSEA for all latent variables (except vitality) were calculated. The CFI and RMSEA could not be calculated for vitality because it contained a small number of observed variables. The criteria used to determine the goodness-of-fit index (GFI) for the whole model was a CFI of ≥0.80 and an RMSEA of ≤0.05. RMSEA values are classified into four categories: close fit (0.00–0.05), fair fit (0.05–0.08), mediocre fit (0.08–0.10), and poor fit (>0.10). In each case, a significance threshold of *p* = 0.10 was set. We used IBM SPSS v.28.0 for Windows (SPSS Japan Inc., Tokyo, Japan) and IBM SPSS Amos v.28.0 (SPSS Japan Inc., Tokyo, Japan) for statistical analysis.

### 2.5. Ethical Considerations

De-identified individual-level CSLC data are available for scientific research upon approval by the Ministry of Health, Labour and Welfare through application procedures under the 1947 Statistics Act, Japan (approval no. 20007010101-25A1; Dr. E. Tadaka). This study was conducted with the approval of the institutional review board of the Medical Department of Yokohama City University (approval no. A210200001).

## 3. Results

**Figure 3:**
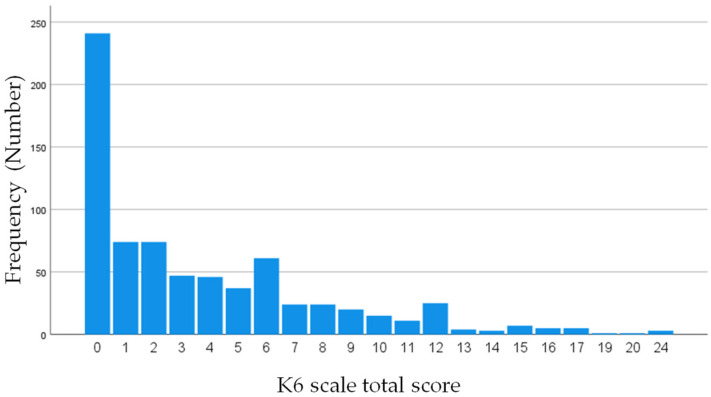
Distribution of adolescents’ six-item Kessler Psychological Distress Scale scores.

The demographic and household environmental variables are shown in Table 1. Overall, 55.9% (n = 407) of the adolescents were girls, 42.6% (n = 310) lived in four-person households, and 91.8% (n = 668) lived in households with employed household heads. The average equivalent household expenditure was 137,000 (SD = 67,000) yen. Household heads had an average K6 total score of 3.8 (SD = 4.4) for mood and anxiety disorders. The school and community environmental variables are shown in Table 2. In total, 66.2% (n = 482) of the adolescents had family support, 54.9% (n = 400) had support from friends, 13.0% (n = 95) had support from teachers, and 4.3% (n = 31) had no social support. Figure 3 shows the adolescents’ K6 scores. The average K6 total score for adolescent mood and anxiety disorders was 3.7 (SD = 4.3). Overall, 33.8% of the adolescents had mood and anxiety disorders. A total of 5.8% (n = 42) of the adolescents had headaches, and 5.6% (n = 41) had fatigue.

We examined the correlations between adolescent mood and anxiety disorders and the other variables in the hypothetical model (Table 3). There was no significant gender difference in mood and anxiety disorders (r = −0.03, *p* = 0.42). Latent variables were determined by the following procedure. First, we excluded somatic symptoms without “headache” and “fatigue” from the analysis because less than 5% of respondents indicated such difficulties. Then, we defined the latent variable “vitality” using two variables because fatigue (*p* < 0.001) and headache (*p* < 0.001) had slightly stronger significant correlations with adolescent mood and anxiety disorders than the other variables. 

Second, household head being employed (*p* = 0.05) and household head having mood and anxiety disorders (*p* < 0.001) were significantly correlated with adolescent mood and anxiety disorders, and single parent household (*p* = 0.048) was significantly correlated with fatigue. We therefore defined the latent variable household environment using these three observed variables (household head employed, household head mood and anxiety disorders, and single parent household) because they were significantly correlated with adolescent mood and anxiety disorders and vitality. Household head having low levels of education and equivalent household expenditure were not significantly associated with adolescent mood and anxiety disorders. However, we included them in the model on a theoretical basis because of previous study findings [23,38,39]. 

Third, the latent variable school and community environment was the summed scores of the three observed variables: family member support, friend support, and teacher support. Friend support and teacher support were not significantly associated with adolescent mood and anxiety disorders, but we included them in the model on a theoretical basis, drawing on results from a previous study [23,39].

**Figure 4:**
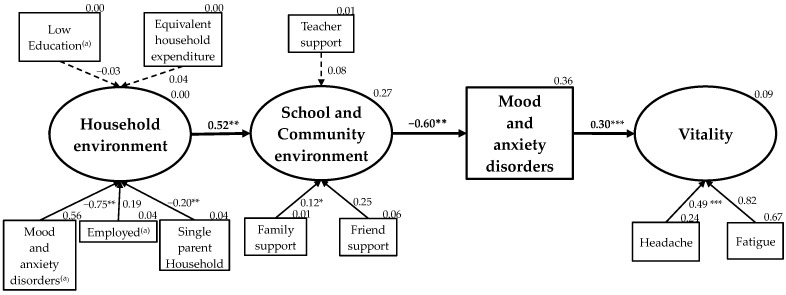
Covariance structure modeling of environments and adolescent mental health (ECO-AM model). Model fit statistics: goodness-of-fit index = 0.984, adjusted goodness-of-fit index = 0.971, comparative fit index = 0.898, root mean square error of approximation = 0.040, the degrees of freedom = 25. Solid lines represent significant standardized path coefficients (* *p* < 0.1, ** *p* < 0.05, *** *p* < 0.001), dotted lines show non-significant standardized path coefficients. Measurement items for mood and anxiety disorders and error variance are not shown. ^(a)^ The household head.

Finally, on the basis of the ecological model, the household environment was modeled as an influencing factor of school and community environment, which were further modeled as an influencing factor of adolescent mood and anxiety disorders and vitality. Figure 4 shows the model and the test results. The fit indices demonstrated that the model fit the data: GFI = 0.956, adjusted GFI = 0.940, CFI = 0.931, and RMSEA = 0.048. The relationships among the factors support the research hypotheses: household environment had a moderate positive effect on school and community environment (β = 0.52, *p* = 0.018); school and community environment had a moderate negative effect on mood and anxiety disorders (β = −0.60, *p* = 0.005); and mood and anxiety disorders had a weak positive effect on vitality (β = 0.30, *p* < 0.001). However, the associations were not significant between household environment and either the household head having low levels of education (β = −0.03, *p* = 0.518) or equivalent household expenditure (β = 0.04, *p* = 0.378). School and community environment and teacher support were also not significantly associated (β = 0.08, *p* = 0.189). The model explained 27% of the variance in school and community environment, 36% of the variance in mood and anxiety disorders, and 9% of the variance in vitality. The fit indices of each latent variable were household environment: CFI = 1.000, RMSEA = 0.097; school and community environment: CFI = 1.000, RMSEA = 0.100; and mood and anxiety disorders: CFI = 0.978, RMSEA = 0.084.

## 4. Discussion

The study aim was to examine an applied ecological model of the sequential association between household, school, and community environmental factors and their effects on adolescent mental health in Japan (ECO-AM model). Data analysis yielded a model with a strong GFI that examined the associations of mood and anxiety disorders and vitality with the household environment and school and community environments in adolescents in Japan.

### 4.1. Model Validity

A unique aspect of this study was the use of data from a large-scale nationwide survey to investigate adolescent mental health. Many studies have examined the mental health of adolescents in Japan, but their findings have limited generalizability because only a few regions and schools have been targeted. To our knowledge, no Japanese studies have used an ecological model to identify inclusive and comprehensive environmental factors associated with adolescent mental health, or have focused on sequential association between the individual and the multilevel environment (household, school, and community environments). There have also been few such studies outside Japan. The studies by Oriol et al. [31] and Rhee et al. [33], which were conducted within the school environment, did not consider the comprehensive framework of the household, school, and community environments. Rhee et al. [33] and Piko et al. [32] measured SES factors such as income, but these studies were not conducted within the inclusive framework of the household environment and did not assess mood and anxiety disorders in household heads. Our model is comprehensive because it focuses on associations between the school, community, and household environments and mood and anxiety disorders and vitality. Our findings therefore provide new information about how the household environment affects the school and community environment, which in turn affects mood and anxiety disorders and vitality in adolescents.

The CSLC is highly representative and uses stratified random sampling of residents across Japan. The valid response rate for this study was high (81.5%). The hypothetical model for this study drew on the results of previous studies. The model (ECO-AM model) showed a strong GFI. We therefore believe that the ECO-AM model is representative of Japanese adolescents who experience worries or stress, and has strong validity from both theoretical and statistical perspectives.

### 4.2. Japanese Adolescent Mood and Anxiety Disorders and Vitality: Strategies to Improve Mental Health and Related Factors

The model derived from the analysis suggested that adolescents experience fewer mood and anxiety problems if they have a supportive school and community environment. The model also indicated a need to improve household environments for adolescents. The selected variables accounted for 36% of the variance in mood and anxiety disorders, demonstrating the importance of the model variables in predicting mood and anxiety disorders.

This model suggested that a better household environment is associated with greater support from the school and community environment. Conversely, adolescents with a fragile household environment may not receive support from the school and community environment. The individual ability of adolescents to use and leverage the resources of their school and community may play a role in this association. Loon et al. [48] demonstrated that parental mental illness affects adolescent internalizing and externalizing of problems, either directly or indirectly through the family environment. In a systematic review of barriers and facilitators of help-seeking behaviors among adolescents aged 10–19 years, trusted and strong relationships with possible gatekeepers (e.g., teachers and parents) and previous positive help-seeking experience facilitated help-seeking behaviors [49]. Another study found that home life and environment were related to poor mental health, less trust, and passive help-seeking from family doctors among students aged 13–17 years in Northern Ireland [50]. This indicates that adolescents who live in households where the household head has low psychological status (e.g., mood and anxiety disorders), or low SES households (e.g., households with single parents or unemployed household heads) may not make good use of school and community resources [50]. Our findings support these previous studies and suggest that improving adolescents’ household environment can reduce internalizing and externalizing of problems, help adolescents to build trust and strong relationships with their parents, friends, and teachers, and increase positive help-seeking experiences. Better household environments may be associated with greater adolescent ability to use and leverage the resources of their school and community environment, thus improving their mental health. Adolescents, particularly those from vulnerable household environments, may therefore need education to enhance their personal abilities and help them to share their concerns. Our model also showed that low levels of education in the household head and equivalent household expenditure do not necessarily affect the school and community environment in adolescents. This may be because equivalent household expenditure was measured by household expenditure rather than household income. This could explain the difference between our findings and those of previous studies on adults [26]. We also did not measure the level of education of the household head in detail (e.g., we were unable to assess the mother’s and father’s education separately). Most previous studies focused on adults, and additional research is therefore required to confirm these findings in adolescents.

Our model also indicated that improving school and community environments can reduce mood and anxiety disorders. These findings support those of previous studies [41,51], which found that low support from family and friends has an effect on adolescent depressive symptoms. Our model also demonstrated that teacher support does not necessarily affect mood and anxiety disorders in adolescents. Mizuta et al. [39] found that teacher support was associated with depression in grade 9 students, and suggested that students’ psychological development may affect this association. In other words, enhancing teacher support alone may not improve the mental health of adolescents.

Our findings also suggest that mood and anxiety disorders in adolescents may be associated with lower vitality. This is consistent with findings from a cross-sectional study that indicated that psychopathology in adolescents was associated with headaches [52], and with those from a longitudinal study, which suggested that depressive symptoms in adolescents predict later increased fatigue [53]. Another cross-sectional study showed that vitality is associated with depressive symptoms [54]. Our model seems to be valid because it explains both theoretical and empirical associations between adolescent mood and anxiety disorders, vitality, and adolescent worries and stress.

These results suggest that future support systems for promoting adolescent mental health should consider household, school, and community environments. Support from friends and family can be effective in reducing mood and anxiety disorders and increasing vitality, particularly in adolescents. Professionals need to recognize that the mental health of caregivers who raise adolescents, and living in a low SES household (e.g., those characterized by single parents and unemployment), affect the mental health of adolescents. It is also important to build support networks that can share information about adolescents at risk of mental health problems; for example, those from low SES households or whose parents have mood and anxiety disorders. Families and schools can perceive the daily mood and anxiety problems and vitality of adolescents. If families, schools, health professionals, and other relevant individuals shared information about the risks to adolescent mental health, and provided appropriate support at an early stage, it might be possible to reduce mood and anxiety disorders and increase vitality. 

These findings provide a new perspective from which to consider the relationship between the household environment and the school and community environment. We believe that the model presented here could provide a basis for developing support network systems to improve adolescent mental health. To ensure that the implementation of this model maintains and/or improves adolescent mood and anxiety disorders and vitality, thus improving mental health, future research needs to focus on two elements: the household environment and the school and community environment. Support network systems are needed to share information about adolescent mental health risks before problems develop, not only within schools but also among doctors, public health nurses, and nurses. If adolescents are provided with effective support, their mental health should improve.

### 4.3. Limitations

This study had five important limitations. First, the cross-sectional research design limited our ability to determine the direction of causation between adolescent environmental variables, mood, anxiety disorders, and vitality. Although the model demonstrated a good fit index, longitudinal research is needed. Second, the inclusion criteria limit the generalizability of the findings, and care should be taken in extrapolating to Japanese adolescents with no stresses or worries. Furthermore, excluding participants who did not state that they felt stressed in the past month would bias the study results. The 1636 adolescents who did not experience distress or stress in the past month (Figure 1) were not obliged to respond to a subsequent survey about the reasons for their distress or stress or where to go for help. Third, a clustering effect may occur in a sample of participants with diverse backgrounds. However, the authors could not control or check for such an effect. Fourth, we were unable to use scales to measure each variable. Furthermore, mood and anxiety disorders were evaluated using self-assessment questionnaires. Finally, the model does not address the interaction of each factor (including bidirectional and cumulative effects). Thus, the possibility of interaction was not investigated, and future analyses should consider interactions.

## 5. Conclusions

The study aim was to examine an applied ecological model of the sequential association between household, school, and community environmental factors and their effects on adolescent mental health in Japan (ECO-AM model). Factors in both the household and school and community environments were related to mood and anxiety disorders and vitality. These findings emphasize the very important role of household environment. We believe that understanding the nature of the household environment will help adolescents seek help from others. Adolescents may not receive support in fragile household environments where the household head is a single parent or has psychological difficulties. Support network systems are needed to share information about adolescent risks before problems develop. Our findings contribute to the understanding of how the household environment affects the school and community environment, which in turn affects mood and anxiety disorders and vitality in adolescents.

## Figures and Tables

**Table 1:** Demographic characteristics of adolescents and their household environment.

			N = 728
		Number	%
Demographic variables			
Gender	Boys	321	44.1
	Girls	407	55.9
Household size (persons)	2	20	2.7
	3	128	17.6
	4	310	42.6
	5	179	24.6
	6 or more persons	91	12.5
Household environment			
Household structure	A couple and unmarried children only	519	71.3
	Three-generation household	110	15.1
	Single parent and unmarried children only	63	8.7
	Other household	36	4.9
Employment status of the household head	Employed	668	91.8
	Not employed	60	8.2
Educational attainment of the household head	Primary or junior high school	68	9.3
	Completion of high school	297	40.8
	Vocational school	77	10.6
	Two-year college/technical college	45	6.2
	University	218	29.9
	Graduate school	23	3.2
Equivalent household expenditure (Yen/A month)		137,000 ± 67,000	(13,000–608,000)
Mood and anxiety disorders of the household head (K6 total score)		3.8 ± 4.4	(0–24)

**Table 2:** Descriptive statistics for school and community environment and mental health.

			N = 728
		Number	%
School and community environment			
Mentor (Multiple answers)	1. Family	482	66.2
	2. Friend	400	54.9
	3. School teacher	95	13.0
	4. Received counseling from a doctor at a hospital or clinic	12	1.6
	5. Used the counseling services of public institutions	6	0.8
	6. Consulted with someone other than the above	4	0.5
	7. I do not consult with anyone because I do not need to consult	86	11.8
	8. I would like to consult but cannot consult with anyone	25	3.4
	9. I would like to consult but do not know where to consult	14	1.9
Recategorized Mentor (Corresponds to the above number)	Family support (1)	482	66.2
	Friend support (2)	400	54.9
	Teacher support (3)	95	13.0
	Other support (One or more of 4 to 6)	20	2.7
	No social support (8 or 9)	31	4.3
Mood and anxiety disorders			
Adolescents’ K6 total score		3.7 ± 4.3	(0–24)
Severity	Standard (K6 total score: 0–4)	482	66.2
	Moderate (K6 total score: 5–13)	217	29.8
	Severe (K6 total score: 14–24)	29	4.0
Vitality			
Somatic symptoms (Multiple answers)	Headache	42	5.8
	Fatigue	41	5.6
	Irritability	27	3.7
	Stomachache	23	3.2
	Dizziness	17	2.3
	Difficulty falling asleep	12	1.6
	Anorexia	6	0.8

**Table 3:** Correlation coefficients of the study variables.

											N = 728
	1.	2.	3.	4.	5.	6.	7.	8.	9.	10.	11.
1. Gender (2 = Girl)	1										
2. Single parent household (1 = Yes)	−0.042	1									
3. Employed ^(a)^ (1 = Yes)	−0.015	−0.014	1								
4. Low education ^(a)^ (1 = Yes)	−0.086 *	−0.015	−0.178 **	1							
5. Equivalent household expenditure (Yen)	−0.061 †	−0.187 **	0.012	−0.130 **	1						
6. Mood and anxiety disorders ^(a)^	−0.060	0.193 **	−0.083 *	−0.003	−0.004	1					
7. Family support (1 = Yes)	0.103 *	0.034	0.092 *	−0.080 *	0.007	−0.032	1				
8. Friend support (1 = Yes)	0.213 **	−0.055	−0.020	−0.041	0.006	0.006	0.129 **	1			
9. Teacher support (1 = Yes)	−0.001	0.026	−0.047	0.002	−0.061 †	−0.046	0.130 **	0.031	1		
10. Fatigue (1 = Yes)	0.001	0.073 *	−0.035	0.024	0.007	0.006	−0.014	−0.042	0.029	1	
11. Headache (1 = Yes)	0.065 †	0.008	−0.076 *	0.002	0.011	−0.002	−0.010	−0.025	0.044	0.400 **	1
12. Mood and anxiety disorders of adolescents	−0.030	0.042	−0.073 *	0.047	0.040	0.265 **	−0.083 *	−0.032	0.024	0.223 **	0.149 **

^(a)^ The household head, † *p* < 0.1, * *p* < 0.05, ** *p* < 0.001.

## Data Availability

Some restrictions apply for availability of data used in this study. Data used for this study are available from the Japan Ministry of Health, Labour and Welfare for researchers who meet the criteria for access to confidential data under the Statistics Act. The address, main phone number, and email address of the ministry are as follows: 1-2-2 Kasumigaseki Chiyoda-ku Tokyo 100-8916, Japan, Tel.: +81-3-5253-1111, Email: nijitekiriyou@mhlw.go.jp. Interested readers are recommended to read Article 33 of the Statistics Act (Act No. 53 in 2007) for eligibility criteria before contacting the Ministry of Health, Labour and Welfare. An English translation of the Statistics Act is available at http://www.soumu.go.jp/english/dgpp_ss/seido/1-1n.htm (accessed on 11 December 2022).
